# The Scope of Metacognitive Therapy in the Treatment of Psychiatric Disorders

**DOI:** 10.7759/cureus.23424

**Published:** 2022-03-23

**Authors:** Vandita Sharma, Rajesh Sagar, Gaurishanker Kaloiya, Manju Mehta

**Affiliations:** 1 Psychiatry, All India Institute of Medical Sciences, New Delhi, New Delhi, IND

**Keywords:** worry, rumination, metacognitive beliefs, cognitive-attentional syndrome, metacognitive therapy

## Abstract

Metacognitive therapy (MCT) is a novel and promising transdiagnostic psychotherapy intervention based on the Self-Regulatory Executive Function model of conceptualizing emotional disorders. It was developed by Adrian Wells in 2009. Its therapeutic response occurs by reducing dysfunctional metacognitive beliefs regarding worry and rumination, often seen in patients with psychiatric disorders. Since its inception, it has been increasingly applied to a wide spectrum of psychiatric illnesses, but mainly focusing on mood and anxiety disorders. To our knowledge, no study has detailed its existing therapeutic scope in psychiatry. In this comprehensive narrative review, we describe the various psychiatric illnesses in which MCT has been used, the advantages of MCT, and the limitations of the MCT research. In addition, we propose some solutions to systematically examine its place in psychiatry. We encountered its potential role in treating trauma and stress-related disorders, obsessive-compulsive spectrum disorders, personality disorders, psychotic disorders, substance use disorders, and sexual disorders.

## Introduction and background

Metacognitive therapy (MCT) [1] is a relatively new therapeutic approach developed for psychiatric disorders. It is framed within Wells and Matthews’ transdiagnostic model of emotional disorders, the Self-Regulatory Executive Function (S-REF) [2]. According to this model, a particular style of repetitive thinking called cognitive-attentional syndrome (CAS) contributes to occurrence, persistence, and relapse in most psychological disorders. CAS comprises an attentional strategy of engaging in internal focus (on thoughts, feelings/emotions, and bodily sensations), ruminating about the past, and worrying about the future along with avoidance behavior and maladaptive mental control strategies that fail and maintain dysfunction [2]. The S-REF model postulates that CAS is supported by negative and positive metacognitive beliefs about these strategies. On one hand, negative metacognitive beliefs take the form of uncontrollability beliefs about rumination, threat monitoring, or worry, for example, a patient may say, “my rumination is uncontrollable.” And on the other hand, positive metacognitive beliefs involve assumptions about the usefulness of rumination and worry, for example, a patient may say “rumination helps me find solutions.” MCT treats psychological disorders by reducing CAS, reshaping metacognitive beliefs, and enabling more adaptive emotional processing among individuals [1]. Its effectiveness has been well tested in patients with major depression and anxiety disorders [3-5]. However, no study has yet reviewed the comprehensiveness of its scope in other psychiatric disorders. In this paper, we provide a broad review of studies on various psychiatric illnesses where MCT has been used as a primary therapeutic intervention. We highlight the potential indications of MCT, its advantages compared to the most popular psychotherapeutic approaches, cognitive behavior therapy (CBT), its limitations, lacunae in research, and the direction research in this field should take in the future.

Methodology

We performed an exhaustive search on PubMed and Google Scholar to find all relevant publications on MCT and various psychiatric disorders that were available till November 2021. We used the following all-embracing keywords for our search: “Metacognitive therapy,” “Metacognitive therapy AND psychiatric disorders,” “Metacognitive therapy AND mental illness,” “Metacognitive therapy AND psychiatric illness,” “Metacognitive therapy AND mental disorders.” Because MCT is well established for depressive and anxiety disorders in the literature, we excluded studies that involved the use of MCT in patients with any of the following: major depressive disorder (MDD), dysthymia, postpartum depression, generalized anxiety disorder (GAD), social anxiety disorder, panic disorder, and phobias as their primary psychiatric diagnosis or when the above-mentioned disorders occurred secondary to a medical/neurologic illness. For this review, we exclusively focused on studies that described the use of MCT based on the manual developed by Adrian Wells [1] for psychiatric disorders and psychological symptoms other than those noted above. Intervention studies that followed manuals and derived their metacognitive interventions from the S-REF model were excluded. Other metacognitively-oriented interventions (metacognitive training [6], metacognition reflection and insight therapy (MERIT) [7], and metacognitive interpersonal therapy [8]) were beyond the scope of this review. We came across studies covering the following psychiatric disorders in the literature through our extensive search: adjustment disorder, prolonged grief disorder, posttraumatic stress disorder (PTSD), obsessive-compulsive disorder (OCD), illness anxiety disorder, body dysmorphic disorder (BDD), binge eating disorder, bipolar disorder, psychosis, sexual disorders, alcohol use disorder, and borderline personality disorder. We also found a few studies in which MCT was used to manage stress and included them in this review, appreciating that stress itself can have numerous negative health consequences. Among studies that we encountered, we included only those that had adult (age ≥18 years) participants. Studies that used only one or some components of the MCT as an intervention or those which used MCT in conjunction with other psychotherapeutic modalities were excluded to highlight the impact of the complete MCT manual (whether administered in a group or an individualized setting) on a given psychiatric disorder. Due to the paucity of research in this domain and for thoroughness, we included case reports, case series, trials (open-label, case-control studies, and randomized controlled trials (RCTs)) in this review. However, we did not include abstracts, conference proceedings, theses, and dissertations. We also did not include studies published in non-English languages.

## Review

Role of the metacognitive therapy in various psychiatric disorders

Adjustment Disorder Secondary to a Physical Illness

Adjustment disorder is common among patients with potentially life-threatening illnesses [9]. According to the Diagnostic and Statistical Manual of Mental Disorders, Fifth Edition (DSM-5), some of the core characteristics of adjustment disorder include preoccupation with the stressor in the form of excessive rumination, worry, and recurrent distressing thoughts. Psychological interventions targeting these thought processes may lead to significant positive outcomes [10]. In a single case study, MCT was found to be helpful in managing symptoms of depression and anxiety in a young woman diagnosed with severe adjustment disorder secondary to pulmonary arterial hypertension [11]. MCT was delivered in an inpatient setting every week for four weeks. At treatment completion, significant reductions in the negative and positive metacognitive beliefs, maladaptive coping strategies, along with improvements in anxiety and depressive symptoms were seen. Because the quality of evidence for psychotherapeutic interventions in adjustment disorder is low, in the future, systematic studies of MCT in managing adjustment disorder will shed more light on the efficacy of this modality.

Prolonged Grief Disorder

PGD is a new diagnostic entity with limited evidence regarding the effectiveness of pharmacological and non-pharmacological interventions [12,13]. It is associated with difficulties in coping with loss, preoccupation with thoughts of the deceased, worry over uncertainty and lack of control over future events, intense distress, high level of self-neglect, avoidance of reminders of loss, and substance use. Its symptomatology is distinct from mood and anxiety disorders and is independently correlated with a significant risk of morbidity and death, suggesting a need to modify the psychotherapeutic strategy to specifically target the underlying psychopathology [12,14]. Because ruminating about the loss and worrying about the uncertainty related to future events are characteristics of PGD, MCT may serve as a promising treatment. The efficacy and feasibility of group MCT for PGD were examined in a pilot study using a randomized waitlist control group design [15]. It was provided in six weekly sessions of two hours each. Twenty-two bereaving adults (MCT = 12, waitlist controls = 10) participated in the study. At post-intervention, in the MCT group, significant improvement was seen in PGD symptomatology, anxiety, depression, stress, rumination, and quality of life, with large effect sizes. Further improvement was observed at the six-month follow-up. This study provided preliminary evidence in support of brief group MCT for PGD, highlighting the need for larger RCTs and comparison studies of MCT for PGD with other psychotherapeutic modalities.

Posttraumatic Stress Disorder

Metacognitive processes such as persistent negative thinking and the use of avoidance coping strategies predict symptoms of PTSD among patients following a traumatic event [16]. The metacognitive model of PTSD [17] proposes that worry and rumination following a traumatic event, threat monitoring, negative metacognitive beliefs about the meanings of symptoms, and avoidance coping styles lock an individual in the experience of trauma and disrupt adaptive processing. One of the first studies on the treatment of PTSD using MCT was performed by Wells and Sembi [18] using a case series design (N = 6). A longer individual follow-up was also carried out for 18-41 months posttreatment. All patients showed large reductions in PTSD symptoms and their severity, depressive symptoms, anxiety symptoms, and emotional distress, with gains remaining stable at the six-month follow-up and in the long-term follow-up. MCT was also effective for treatment-resistant PTSD in a case study [19]. The scope of MCT in PTSD was further expanded in an open trial (N = 11) by evaluating its efficacy in chronic PTSD [20]. Clinically significant recovery was observed among 55.5% of the participants, while 33.3% of the participants showed reliable improvement at the six-month follow-up. After obtaining supportive evidence for the effectiveness of MCT in PTSD, Wells and Colbear (2012) [21] performed an RCT to examine the efficacy of MCT in comparison to waitlist controls among patients with chronic PTSD. Twenty participants with chronic PTSD were randomly allocated to the MCT group (N = 10) or the waitlist control group (N = 10). Significantly greater improvements were obtained in the MCT group compared to the control group (per intent-to-treat (ITT) analysis) in PTSD symptoms, depressive symptoms, anxiety symptoms, emotional distress caused by traumatic events, and worry. At the six-month follow-up, up to 80% of the participants in the MCT group had achieved clinical recovery. This study bolstered the cumulating evidence of the efficacy of MCT in PTSD. MCT has also been compared to evidence-based manualized prolonged exposure therapy (PE) and waitlist controls (WL) in an RCT in patients with chronic PTSD [22]. Patients with moderate-to-severe chronic PTSD were randomly assigned to one of the three groups: MCT (N = 11), PE (N = 11), and WL (N = 10). At posttreatment, the MCT group experienced a significantly greater and faster reduction in PTSD symptoms and physiological arousal (heart rate) compared to the PE group. Clinically significant recovery in PTSD symptoms was seen among all participants in the MCT group and 70% of the participants in the PE group at post-treatment.

To summarize, there is burgeoning evidence regarding the benefits of MCT in PTSD. However, the psychological mechanisms underpinning the improvement brought by MCT in these patients have not been investigated yet. More studies are needed with a focus on outcome measures that explore the change in metacognitive beliefs, thought control strategies, and coping styles commonly used by patients with PTSD. Future trials should implement larger sample sizes and longer follow-up periods to assess the long-term success of MCT in PTSD. The effectiveness of MCT compared to other psychotherapeutic approaches traditionally used in the management of PTSD remains to be established. Future studies should also focus on the application of MCT in more complex trauma and/or different types of traumas.

Obsessive-Compulsive Disorder

According to the metacognitive model of OCD, the three key factors that are important in the development of OCD include the importance that an individual assigns to commonly occurring obsessional intrusive experiences, the strategies of self-regulation that the individual uses to process these experiences, and the experiential perspective that they take in relation to these experiences [1,23]. The three types of metacognitive beliefs associated with OCD include thought-event fusion beliefs, thought-action fusion beliefs, and thought-object fusion beliefs [23]. These metacognitive beliefs lead to a negative interpretation of the intrusive events, heightened threat monitoring, and resulting anxiety. One of the first studies examining the potential of MCT in treating OCD used a case-series design among four patients with OCD [24]. Following treatment, 63-75% reduction in OCD symptoms based on the Yale-Brown Obsessive-Compulsive Scale (Y-BOCS) was observed for all patients, along with improvement in the frequency of OCD symptoms, depressive symptoms, anxiety symptoms, and metacognitive beliefs about the intrusive thoughts, obsessions, and compulsions. At the six-month follow-up, two patients remained recovered. The effectiveness of MCT was also compared with fluvoxamine among patients with OCD [25]. Nineteen participants with OCD were randomly assigned to one of the three treatment conditions: MCT alone (N = 7), fluvoxamine alone (N = 6), and MCT with fluvoxamine (N = 6). At post-intervention, all participants in the MCT group recovered on Y-BOCS compared to about 84% of participants in the combined treatment group. Participants receiving combined treatment had significant improvement in OCD symptoms compared to those in the fluvoxamine group. Overall, MCT, when offered alone, or in combination with pharmacotherapy was more effective compared to pharmacotherapy alone. The efficacy of MCT has also been demonstrated in patients experiencing obsessions without overt compulsions [26] in a case series. A relatively large open-label trial was conducted by Van der Heiden et al. (2016) [27] including 25 participants with OCD. Among study completers (N = 19), MCT was associated with large effect sizes for obsessive-compulsive (OC) symptoms, depressive symptoms, and thought-fusion beliefs at the three-month follow-up. Similar findings were also reported in a case study examining the efficacy of MCT in managing OC symptoms in a middle-aged individual [28]. Treatment was associated with large reductions in dysfunctional beliefs about OC symptoms and clinically significant recovery on Y-BOCS, with gains maintained at the three-month follow-up. A naturalistic study by Papageorgiou et al. (2018) [29] compared group CBT (n = 125) with group MCT (n = 95) in a cohort of patients with OCD. Around 86% of patients who received group MCT responded compared to only 64% of those who were administered group CBT. Some notable study limitations were a lack of treatment fidelity and adherence evaluation, absence of independent raters, and lack of accounting for the type of medications received by the patients.

More recently, attempts have been made to compare the effectiveness of MCT for OCD against other psychological interventions. In a pilot RCT, the effectiveness of MCT was compared with ERP among patients with OCD [30]. The study participants were randomly assigned to the MCT group (N = 19) and the ERP group (N = 18). Both MCT and ERP led to significant improvements. Participants in the MCT group needed less time with the therapist compared to the ERP group. Thus, MCT for OCD may be equally effective and less time-consuming in comparison to ERP.

Overall, these findings suggest that MCT is an efficacious treatment that can be offered over a brief duration to manage OC symptoms.

Illness Anxiety Disorder

Illness anxiety disorder is characterized by persistent and excessive preoccupation and fear associated with the belief that one has or could acquire a serious illness, as well as high levels of anxiety about health [31]. It has recently been revised from hypochondriasis to illness anxiety disorder by both DSM-5 and International Classification of Diseases 11th Revision [32]. So far, CBT is the most well-researched psychotherapy intervention for this disease [33]. However, a recent meta-analysis showed that only about 50% of the patients receiving CBT achieved remission at post-intervention and one-third of the patients did not respond [34]. Given the low remission and response rates with CBT, MCT may be an encouraging new treatment to address the psychopathology underlying illness anxiety. The application of specific techniques of MCT as a stand-alone treatment in managing illness anxiety disorder has shown good results [35,36]. We found one case series on the effectiveness of MCT in patients with illness anxiety disorder [37]. All patients had salient improvements in depressive and anxiety symptoms, as well as a significant reduction in dysfunctional metacognitive beliefs at even the six-month assessment.

Body Dysmorphic Disorder

BDD is characterized by excessive and persistent preoccupation with perceived defects or flaws in one’s appearance [38]. It is associated with a high risk of suicidality and psychiatric comorbidity [39]. The most efficacious and recommended treatments for BDD include CBT (using ERP) and selective serotonin reuptake inhibitors (SSRIs) [40]. RCTs have shown that CBT has a large effect size in reducing BDD symptoms, but data is limited regarding its long-term effects [41]. Further, the response rates of CBT in BDD range from 40% to 54% [41]. The role of metacognition in relation to BDD symptoms has been established in previous studies [42,43]. The effectiveness of MCT in BDD has been examined in one RCT [44]. This study included 20 participants with BDD. Participants in the experimental group (N = 10) received eight sessions of MCT, whereas the waitlist control group (N = 10) received no intervention. Posttreatment, the MCT group had the largest improvements in BDD symptoms and metacognitive beliefs about the meaning, significance, and hazards of intrusive thoughts, while the waitlist group had no change. Treatment response with MCT remained stable during the six-month follow-up period. Clinically significant recovery was observed among 70% of the participants in the MCT group at posttreatment and 60% at the six-month follow-up. Future research should focus on comparative studies of MCT with CBT and other evidence-based psychotherapies to examine which therapies would be the most effective in the management of BDD.

Eating Disorders

The current understanding of the possible role of MCT in eating disorders is very limited. Disordered eating may be associated with perseverative thinking, dysfunctional metacognitive beliefs, and attentional biases [45,46]. Despite existing established evidence on the significance of metacognitive beliefs in anorexia nervosa and bulimia nervosa, no published research was found on the role of MCT in these two diseases. We found a case series on the use of MCT in binge eating disorders [47] which used a general metacognitive model for eating disorders [48]. The intervention was based on the Wells’ manual [1], the MCT formulation developed by Vann et al. (2013) [48], and an unpublished therapist’s manual [49]. At post-intervention and follow-up assessment, all participants had recovered. MCT was also associated with a clinically significant reduction in anxiety and depressive symptoms and improvements in negative and positive metacognitive beliefs, cognitive confidence, and metacognitive coping strategies. Because the intervention used here did not strictly adhere to Wells’ manual [1], it is difficult to comment on the benefit of MCT in binge eating disorders. More studies are required to identify the role dysfunctional metacognition plays in patients with eating disorders and to assess the effectiveness of MCT in the treatment of anorexia nervosa, bulimia nervosa, and binge eating disorder.

Bipolar Disorder

The effectiveness of MCT in the management of bipolar disorder is yet to be established. One published case series examined its role in treating bipolar disorder type II [50]. At treatment completion, no patient met the diagnostic criteria for a major depressive episode. Systematic studies are required to examine the effectiveness of MCT in bipolar disorder.

Psychosis

The standard and most widely used treatment of psychosis is pharmacotherapy [51]. However, a substantial number of patients continue to experience negative symptoms and cognitive and social impairments, despite adequate antipsychotic trials [52]. Augmenting pharmacological treatment of schizophrenia with psychosocial interventions may help in improving overall treatment outcomes [53]. Meta-analytic studies have found that CBT has a moderate effect size in improving symptoms among patients with treatment-resistant psychosis [54]. Newer treatment approaches that go beyond the scope of cognitive theories in the treatment of psychosis have been developed [55]. It has been demonstrated that metacognitive processes may also contribute to the development and maintenance of psychosis [56]. One of the earliest suggestions for the role of MCT in the treatment of psychosis was found in a case series of patients with treatment-resistant schizophrenia [57]. The treatment used general guidelines of MCT for GAD [1]. Two out of three participants had a significant decrease in delusions, depressive symptoms, and anxiety symptoms at posttreatment compared to baseline. Because the two participants also had changes in their medications during the study period, the improvements could not be clearly attributed to MCT. The researchers recruited additional (seven) participants hoping to strengthen their results [58]. Statistically significant and large reductions were seen in the positive symptoms and delusions at posttreatment compared to baseline. Improvements in positive symptoms remained statistically significant compared to baseline. However, improvement in delusions did not sustain during follow-up. No significant improvements were noted for depressive symptoms, anxiety symptoms, social functioning, self-rated recovery, and most metacognitive domains at posttreatment and follow-up. In summary, MCT was feasible and effective in managing positive symptoms and delusional beliefs related to psychotic illness in the short term. The feasibility of MCT has also been examined among individuals at high risk for psychosis. In a recent open-label trial, Parker et al. (2020) [59] examined the feasibility and acceptability of MCT in 10 individuals at ultra-high risk of developing psychosis. All participants were assessed for “at-risk” symptoms; appraisals of hearing voices; personal, social, and psychological functioning; depressive and anxiety symptoms; CAS activity; metacognitive beliefs; and metacognitive beliefs about paranoia. At post-intervention, statistically significant improvements were observed in some psychotic-like experiences; personal, social, and psychological functioning; depression and anxiety symptoms; worry and threat monitoring; and appraisals of hearing voices. Statistically significant improvements were also observed in negative metacognitive beliefs. At the six-month follow-up assessment, improvements remained statistically significant for non-bizarre ideas. Improvements in anxiety symptoms and appraisals of hearing voices also remained statistically significant at follow-up, in addition to changes in metacognitive beliefs. However, improvements in other psychotic-like experiences seen post-intervention did not survive in the follow-up period. Four participants no longer met the criteria for “at-risk mental state” at follow-up. In summary, MCT appears to be effective in reducing some psychotic-like experiences, anxiety, and depression among individuals at ultra-high risk of developing psychosis.

Overall, it can be concluded that MCT is a feasible and acceptable treatment in managing psychotic illnesses. It has shown promising results in managing positive symptoms including delusions and general psychiatric symptoms of schizophrenia among patients with treatment-resistant psychosis in the short term. The feasibility of MCT has also been established in reducing psychotic-like experiences and improving overall functioning among individuals at high risk of developing psychosis. Future studies should focus on examining the effectiveness of MCT among patients with psychosis using larger samples and rigorous methodological design.

Borderline Personality Disorder

MCT has also been applied in the management of BPD. MCT for BPD has three main components [60]. The first component includes negotiating a contract with the patient and shaping their expectations about the intervention. The next component targets modifying the self-defeating beliefs and self-regulatory executive functions. The final component aims at providing general psychiatric management involving community psychiatry services. Nordahl and Wells (2019) [60] examined the effectiveness of MCT for the treatment of BPD in 12 patients with a history of severe early childhood sexual abuse. Using a baseline-controlled design, up to 40 sessions of MCT (range: 20-45) were provided to the participants over one year. No participant dropped out, and all participants attended 70-90% of sessions. Significant improvements were seen in BPD-related symptoms, depression, anxiety, posttraumatic symptoms, quality of life, suicidal thoughts, self-harming behaviors, rumination and worry about abandonment and rejection, and interpersonal dysfunction, with gains maintained at the two-year follow-up. This suggests that MCT is feasible for outpatient treatment and associated with good clinical outcomes in patients with BPD.

Alcohol Use Disorder

The S-REF model of psychopathology has also been applied in the conceptualization and treatment of alcohol use disorder [61,62]. According to this model, dysfunctional metacognitive beliefs, perseverative thinking, ineffective metacognitive monitoring, and impairment in attentional functioning have been linked to alcohol use disorder. One of the techniques of MCT has been shown to be more effective as a stand-alone treatment vis-à-vis brief exposure exercise in reducing distress and urge to use alcohol in a small sample of patients [63]. MCT has been found to help achieve controlled or reduced-risk alcohol use among patients with alcohol use disorder in a recent case series [64]. Posttreatment, a significant reduction in self-reported weekly alcohol use was observed in all patients which was maintained at the six-month follow-up. No patient engaged in binge drinking during follow-up. Significant reductions in dysfunctional metacognitive beliefs related to alcohol use, anxiety, depression, and alcohol cravings were also seen. Three patients achieved clinically significant recovery, while two met the criteria for clinically significant improvement at the six-month follow-up.

Sexual Disorders

There is no MCT model described for sexual disorders. A novel study by Ramezani et al. (2017) [65] studied the impact of MCT (using the depression model of MCT proposed by Wells, 2009 [1]) in a multi-center, RCT (n = 30) among patients with hypoactive sexual desire disorder. The intervention arm received MCT, and the control arm was administered Masters-Johnson Sex Therapy (MJST). The MCT group outperformed the MJST group at post-intervention assessment. Unfortunately, both groups lost some efficacy at the six-month follow-up. The between-group difference on the primary outcome measure was no longer significant at the six-month follow-up. One of the major weaknesses of this study was the non-inclusion of metacognitive beliefs and factors such as worry and rumination in the assessment, precluding a mechanistic understanding of the results.

Role of the metacognitive therapy in managing psychological symptoms

Work-Related Stress

A recent feasibility study by de Dominicis et al. (2021) tested the impact of MCT on managing chronic work-related stress among four individuals [66]. Only participants without any psychiatric illness and not receiving any psychotherapy in the last two years were chosen. All participants had to be on sick leave due to work-related stress with no symptom recovery. General mental health, perceived stress, and blood pressure were chosen as primary outcome variables. MCT protocol for GAD was used [1]. All participants went back to work after treatment completion and continued working during follow-up. Maladaptive coping, avoidance behaviors, and depression symptoms improved as well. The gains were maintained till the six-month follow-up.

We propose that a metacognitive model for stress should be developed, and larger randomized controlled studies are required to fully determine the efficacy of MCT on work-related stress.

Emotional Distress Due to Cancer

Fisher et al. (2017) [67] studied the effect of MCT to reduce distress among four cancer survivors consecutively referred to a psycho-oncology service. Participants’ anxiety, depression, worry/rumination, fear of cancer recurrence, and metacognitive beliefs were assessed. Primary outcomes used were anxiety and depressive symptoms and time spent worrying/ruminating. All participants achieved significant reductions in all the psychological constructs mentioned above at the three-month follow-up. Three participants remained improved at the six-month assessment as well.

Buoyed by the above study’s success, Fisher et al. (2019) [68] conducted an open-label trial (N = 27) of MCT among consecutive cancer survivors referred to a psycho-oncology service. Significant improvement was again noted across all outcome measures, with improvement sustained at the six-month follow-up. ITT analysis found that 52% of the participants recovered at the six-month follow-up. Major shortcomings of this study were a lack of data on treatment adherence or therapist competence and the use of only self-reported measures of treatment response.

Discussion

MCT has begun creating an impact on the treatment of a wide variety of psychiatric disorders worldwide. It is making ground in the treatment of major depression and anxiety disorders, two areas of research where most of its thrust had been directed till now [3-5,69-75]. Owing to its transdiagnostic approach, which makes it easily adaptable, and requires less time capital to gain mastery, it may naturally appeal to psychotherapists. It is well placed in relation to the existing realities of psychiatric disorders where comorbidity is the rule rather than the exception, and often comorbid disorders have similar underlying psychological substrate and psychosocial vulnerabilities [76-78]. Its principles can be applied to treat symptoms of multiple psychiatric disorders concurrently instead of treating different psychiatric disorders sequentially with approaches such as CBT. It is also shorter in duration than CBT, which often requires 12-20 sessions [79-81]. Thus, it may bring improvement quickly, decrease disease-related distress and dysfunction, and reduce the overall cost of the treatment while freeing up the therapist’s time, enabling them to care for more patients. So far, it has not only been found to be feasible and acceptable (evidenced by high treatment adherence) but has also shown a robust response in treating various psychiatric disorders. Mounting evidence supports the use of MCT in a host of psychiatric disorders, both internalizing and externalizing; mood disorders, anxiety spectrum disorders, OCDs, trauma and stress-related disorders, psychotic disorders, eating disorders, sexual disorders, and substance use disorders. It may even be helpful in managing stress due to psychosocial stressors (work-related) and life-threatening medical illnesses (such as cancer) (See Table 1 for a summary of the studies included in this review). Emerging research also suggests that MCT has been highly effective in managing mood and anxiety symptoms in patients with cardiovascular diseases [77]. These wide-spectrum indications have the potential to elevate the status of MCT as equal to or perhaps greater than CBT in due course of time. The key limitations of MCT research studies are practically non-existent RCTs, lack of control group in the available studies, and short follow-up duration, moderating our confidence in the efficacy of MCT. Most studies have recruited a very small number of patients and have lacked blinding, thus limiting the generalizability of their results. Curiously, most studies [18,21,24,60,74,75,77,82-86] have been published by the same group of researchers who developed MCT, thereby raising concerns of biased results. At present, there exist vast open spaces in the MCT research field, especially in terms of its mechanism of action, its comparison to well-established treatments such as CBT, population groups in whom it may be applicable, considering that it works on metacognition, which may not be well developed (e.g., children, people with cognitive disorders) [87-92], the durability of response, whether it shows a synergistic effect with pharmacological treatments, and its effect on neurobiology. It would be interesting to study if it will be effective in patients with no or minimal education, and whether its efficacy differs according to sociodemographic settings. Most studies using MCT have had very strict selection criteria; therefore, it is not clear how effective it will be in the real-world setting, making a case for conducting studies with liberal selection criteria.

**Table 1:** MCT in various psychiatric disorders.

Author	Disorder	Number of sessions	Sample size	Sex	Study design	Outcome measures	Key findings
Winter et al., 2020 [11]	Adjustment disorder	4	1	F	Case study	HADS, MCQ-30	Significant reduction in positive and negative metacognitive beliefs, anxiety, and depressive symptoms. Gains stable at the six-week f/u
Callesen et al., 2020 [50]	Bipolar disorder	7–12	3	1 F, 2 M	Case series	BDI-II, YMRS, CAS-1	All patients achieved remission at f/u, reductions in time spent on rumination, and metacognitive beliefs
Wenn et al., 2019 [15]	PGD	6	22	21 F, 1 M	RCT: MCT vs. WL	PG-13, DASS-21, UGRS, MCQ-30, Q-LES-Q-18, CGI	At post-intervention, the MCT group showed improvement in PGD symptoms, depression, anxiety, stress, rumination, and QoL. Gains maintained at the three-month f/u, and further improvement at the six-month f/u. Similar findings in treated controls
Robertson and Strodl, 2020 [47]	Binge eating disorder	12	3	3 F	Case series	BES, EOQ, TCQ, MCQ-30, BDI-II, BAI	At post-intervention and f/u assessment, all patients achieved remission, improvement in depressive and anxiety symptoms, metacognitive beliefs, cognitive confidence, and metacognitive coping strategies
Bailey and Wells, 2014 [37]	Illness anxiety disorder	6–9	4	3 F, 1 M	Multiple baseline case series	WI, MCHQ, BAI, BDI-II, MCQ-30	All patients recovered at post-treatment and the six-month f/u. At post-treatment, improvements in anxiety, depressive symptoms, metacognitive beliefs, and gains maintained at the six-month f/u
Rabiei et al., 2012 [44]	BDD	8	20	18 F, 2 M	Uncontrolled trial	BDD-YBOCS, TFI	At post-treatment, improvement in BDD symptoms, metacognitive beliefs in the MCT group, no change in waitlist controls. Gains maintained at the six-month f/u. Recovery in 70% participants in the MCT group at post-treatment, and 60% at the six-month f/u
Nordhal and Wells 2019 [60]	Borderline personality disorder	Up to 40 (20–45)	12	10 F, 2 M	Multiple baseline case series	IIP-64, PDS, ERIS, WHO-5, BDI-II, BAI, SCID-II criteria for BPD	Improvement in borderline personality-related symptoms, depression, anxiety, posttraumatic symptoms, QoL, suicidal thoughts, self-harm behaviors, rumination and worry, and interpersonal dysfunction. Gains maintained at the one-year and two-year f/u
Caselli et al., 2018 [64]	Alcohol use disorder	12	5	5 M	Non-concurrent multiple baseline case series	AUDIT-C, HADS, PAMS, NAMS, PACS, QFS, CAS-A	Significant reduction in weekly alcohol use, binge drinking, craving, dysfunctional metacognitive beliefs, depression, and anxiety symptoms. Gains maintained at the three-month and six-month f/u
Wells and Sembi, 2004 [18]	PTSD	8	6	5 F, 1 M	Multiple baseline case series	DTS, IES, PI, BAI, BDI, PDS	Reductions in PTSD symptoms and severity, anxiety symptoms, depressive symptoms, emotional distress at posttreatment. Gains maintained at the three-month and six-month f/u and long-term f/u between 18 and 41 months
Vakili and Fata, 2006 [19]	PTSD	8	1	1 M	Multiple baseline case study	IES-R, BDI-II, BAI, SUDS	Large reductions in PTSD symptoms, anxiety symptoms, depressive symptoms, and emotional distress. Gains maintained at the one-month, three-month, and six-month f/u
Wells et al., 2008 [20]	PTSD	3–15 (m = 8.5)	11	6 F, 5 M	Open-label trial	IES, PI, BAI, BDI	Statistically significant improvements with large effect size at post-intervention in PTSD symptom severity, depressive symptoms, anxiety symptoms, and emotional distress. Similar effect sizes at the three-month and six-month f/u for outcome measures. Clinically significant improvement in one-third of the participants and recovery in ~55% of the participants at the six-month f/u
Wells and Colbear, 2012 [21]	PTSD	8 (m = 6.4)	20	11 F, 9 M	RCT: MCT vs. WL	PDS, IES, BDI-II, BAI, TCQ	Significantly greater improvements in the MCT group compared to the control group at posttreatment in PTSD symptoms, anxiety symptoms, depressive symptoms, emotional distress, and worry severity, with large effect sizes. Improvements maintained within the MCT group at the three-month and six-month f/u. Clinically significant recovery in 60–80% of the participants within the MCT group at six-month f/u
Wells et al., 2015 [22]	PTSD	8	32	12 F, 20 M	Triple-arm RCT: MCT vs PE vs. WL	PDS, IES, BDI-II, BAI, Heart rate	Both MCT and PE > WL, MCT > PE in reducing symptoms of PTSD and physiological arousal
Fisher & Wells, 2008 [24]	OCD	12	4	2 F, 2 M	Case series	Y-BOCS, PI, BDI, BAI, MOCI, TFI, OCBQ	Large percentage reductions in the frequency and severity of OCD symptoms, depressive symptoms, anxiety symptoms, metacognitive beliefs related to obsessions and compulsions. All patients met the clinical recovery criteria on Y-BOCS at post-intervention and the three-month f/u. Two out of four patients continued to remain recovered at the six-month f/u
Shareh et al., 2010 [25]	OCD	10	19	10 F, 9 M	RCT: MCT vs. fluvoxamine vs. MCT + Fluvoxamine	Y-BOCS, BDI-II, BAI	Significant differences between three groups at posttreatment, suggesting superior gains in the MCT group, followed by the combination group, and the fluvoxamine group
Andouz et al., 2012 [26]	OCD	14	6	4 F, 2 M	Multiple baseline case series	SCID-I, OCI-R, Y-BOCS, MCQ-30, TFI, BDI-II	Significant and large percentage reductions in obsessive-compulsive symptoms, depressive symptoms, thought-fusion beliefs, and dysfunctional metacognitive beliefs at posttreatment. Further improvements in each of these outcome variables was seen at the three-month f/u
Van der Heiden et al., 2016 [27]	OCD	Up to 15 (m = 13.7)	25	17 F, 8 M	Uncontrolled single group trial	Padua inventory, Y-BOCS, BDI-II, TFI,	Large effect sizes for obsessive-compulsive symptoms, depressive symptoms, thought fusion beliefs at posttreatment and three-month f/u for completers sample. Large effect sizes for Y-BOCS in the ITT sample
Melchior et al., 2018 [28]	OCD		1	M	Case study	Padua inventory, Y-BOCS, SCID-I, TFI, BARI	Clinically significant recovery on the Y-BOCS, large reductions in dysfunctional beliefs about symptoms. Gains maintained at the three-month f/u
Papageorgiou et al., 2018 [29]	OCD	12 (two-hourly)	Group CBT = 125 Group MCT = 95	Group CBT, M/F (59/66) Group MCT M/F (50/45)	Comparative study	Y-BOCS, BDI, WSAS, SRGIS	The MCT group produced better outcomes in an ITT analysis. Around 86% of the patients who received group MCT responded compared to only 64% of those who were administered group CBT
Glombiewski et al., 2021 [30]	OCD	10–14	37	24 F, 13 M	RCT: MCT vs. ERP	Padua Inventory revised, Y-BOCS, MCQ, BDI-II, Credibility/Expectancy questionnaire	No significant between group differences on Y-BOCS at posttreatment and f/u. Significant differences in the time spent with the therapist between the MCT and ERP groups. Significant between-group differences in the need for further treatment at the end of the treatment
Hutton et al., 2014 [57]	Treatment-resistant psychosis	11–13	3	2 F, 1 M	Multiple baseline case series	PSYRATS, PANSS, BDI-II, BAI, QPR, CAS-1	Large reductions in delusion symptoms, depressive symptoms, and anxiety symptoms for 2/3 participants at posttreatment compared to baseline. Improvements not sustained during f/u. Reduction in CAS activity at posttreatment in two participants
Morrison et al., 2014 [58]	Treatment-resistant psychosis	up to 12 (m = 10.6)	10	2 F, 8 M	Case series	PANSS, PSYRATS, QPR, BDI, BAI, MCQ-30, PSP	Reductions in positive symptoms, and total scores on PANSS and delusion symptoms on PSYRATS at posttreatment. Improvement in delusional symptoms lost during f/u. No improvements in depressive symptoms, anxiety symptoms, social functioning, and self-rated recovery at posttreatment and f/u
Parker et al., 2020 [59]	Individuals at high risk of psychosis	Up to 12 (m = 8)	10	4 F, 6 M	Case series	CAARMS, IVI, HADS, GAF, MCQ-30, CAS-1, BAPS	Improvements at post-intervention in psychotic-like experiences, personal, social, and psychological functioning, depression and anxiety symptoms, CAS activity, appraisals of hearing voices, negative metacognitive beliefs, need to control thoughts, cognitive confidence. Few improvements in psychotic-like experiences maintained at the six-month f/u. Improvements in anxiety symptoms, appraisals of hearing voices, negative metacognitive beliefs, need to control thoughts, and cognitive confidence maintained at f/u
Ramezani et al., 2018 [65]	Hypoactive sexual desire disorder	10 sessions of MCT 10 sessions of MJST	30	23 F, 7 M	RCT: MCT vs. MJST	FSFI, GHQ-28, ENRICH	MCT outperformed the MJST group at post-intervention, latter did not show any improvement. Both groups had score reduction at the six-month f/u losing significance
de Dominicis et al. 2021 [66]	Work-related stress	8–10	4	3 F, 1 M	Multiple baseline Case series	GHQ-30, PSS-10, GADS-R, BDI-II, SCID-I	All participants went back to work, maladaptive coping strategies, avoidance behaviors, and depression symptoms improved. Gains maintained at the six-month f/u
Fisher et al., 2017 [67]	Cancer-related emotional distress	6	4	4 F	Multiple baseline case series	HADS CAS-1, FCRI, MCQ-30	All participants had reductions in symptoms at treatment end, gains maintained at the three-month f/u. Three remained improved at the six-month f/u
Fisher et al., 2019 [68]	Cancer-related emotional distress	6	27	23 F, 4 M	Open-label trial	HADS, IES-R, FCRI, FACT-G, MCQ-30, and CAS-1	Improvement noted across all outcome measures, gains maintained at the six-month f/u. ITT found 52% of participants recovered at the six-month f//u
f/u = follow-up; F = female; M = male; m = mean; HADS = Hospital Anxiety and Depression Scale; MCQ-30 = Metacognitions Questionnaire-30; BDI-II = Beck Depression Inventory-II; YMRS = Young Mania Rating Scale; CAS-1 = Cognitive-Attentional Syndrome Scale-1; PG-13 = Prolonged Grief Disorder-13; DASS-21 = Depression Anxiety and Stress Scale; UGRS = Utrecht Grief Rumination Scale; Q-LES-Q-18 = Quality of Life Enjoyment and Satisfaction Questionnaire; CGI = Clinical Global Impressions Scale; BES = Binge Eating Scale; EOQ = Emotional Overeating Questionnaire; TCQ = Thought Control Questionnaire; BAI = Beck Anxiety Inventory; WI = Whiteley Index; MCHQ = Metacognitions About Health Questionnaire; BDD-YBOCS = Yale-Brown Obsessive Compulsive Scale Modified for Body Dysmorphic Disorder; TFI = Thought Fusion Instrument; IIP-64 = The Inventory of Interpersonal Problems; PDS = Post-traumatic Stress Diagnostic Scale; ERIS = Emotional and Relationship Instability Scale; WHO-5 = WHO-5 Wellbeing Index; SCID-II = Structured Clinical Interview for DSM-IV Axis II Personality Disorders; BPD = borderline personality disorder; AUDIT-C = Alcohol Use Disorders Identification Test Consumption; PAMS = Positive Alcohol Metacognitions Scale; NAMS = Negative Alcohol Metacognitions Scale; PACS = Penn Alcohol Craving Scale; QFS = Quantity Frequency Scale; CAS-A = Cognitive Attentional Scale – Alcohol; DTS = Davidson Trauma Scale; IES = Impact of Events Scale; PI = Penn Inventory for Post-traumatic Stress Disorder; BDI = Beck Depression Inventory; PDS = Posttraumatic Stress Diagnostic Scale; IES-R = Impact Event Scale-Revised; SUDS = Subjective Units Distress Scale; Y-BOCS = Yale-Brown Obsessive Compulsive Scale; PI = Padua Inventory; MOCI = Maudsley Obsessive–Compulsive Inventory; OCBQ = Obsessive-Compulsive Beliefs Questionnaire; SCID-I = Structured Clinical Interview for DSM-IV Axis I Disorders; OCI-R = Obsessive Compulsive Inventory (Revised Form); BARI = Beliefs About Rituals Inventory; WSAS = Work and Social Adjustment Scale; SRGIS = Self-Ratings of Global Improvement Scale; PSYRATS = Psychotic Symptom Rating Scales; PANSS = Positive and Negative Syndrome Scale; QPR = Questionnaire About the Process of Recovery; PSP = Personal and Social Performance Scale; CAARMS = Comprehensive Assessment of At-Risk Mental States Interview; IVI = Interpretations of Voices Inventory; GAF = Global Assessment of Functioning; BAPS = Beliefs About Paranoia Scale-Short Form; FSFI = Female Sexual Function Index; GHQ-28 = General Health Questionnaire; ENRICH = Evaluation & Nurturing Relationship Issues Communication and Happiness Questionnaire; GHQ-30 = General Health Questionnaire; PSS-10 = Perceived Stress Scale; GADS-R = Generalized Anxiety Disorder Scale-Revised; FCRI = Fear of Cancer Recurrence Inventory; FACT-G = Functional Assessment of Cancer Therapy-General							

## Conclusions

To summarize, MCT appears quite encouraging in its ability to deliver across a gamut of psychiatric disorders. Treatment using MCT has led to remission of various psychiatric symptoms including OCD, PTSD, illness anxiety disorder, BDD, adjustment disorder due to physical illness, prolonged grief disorder, binge eating, binge drinking, hypoactive sexual desire, depressive episodes in bipolar affective disorder, and positive symptoms and delusional beliefs among patients with psychosis. Gains obtained through treatment remain stable for relatively longer durations (up to one year). Moreover, MCT is highly efficacious in reducing emotional distress experienced during chronic life-threatening illnesses such as cancer, as well as in day-to-day work-related stress. In the future, we encourage researchers at different centers globally to conduct replication studies of MCT for the psychiatric disorders discussed in this review and to test its effectiveness in other psychiatric disorders not investigated so far. Researchers should intend to recruit larger patient samples, use rigorous methodological designs (blinding, control group, randomization, real-world setting, comparison with established psychotherapeutic modalities such as CBT), and employ longer follow-up duration. This exercise may prove to be quite rewarding because our field is grappling with a crisis of unsatisfactory and inadequate treatments for most psychiatric disorders.
